# Supplementary material for: Matching Habitat Choice and the Evolution of a Species’ Range
Source: Bull Math Biol. 2025 May 7;87(6):70. doi: 10.1007/s11538-025-01445-x (PMC12058903; doi:10.1007/s11538-025-01445-x)
Supplement: Supplementary file 1 — (pdf 2143 KB) [file 11538_2025_1445_MOESM1_ESM.pdf]

Matching Habitat Choice and the Evolution of a Species' Range

(Supplementary Figures)

Farshad Shirani[1*] and Judith R. Miller[2]

[1*]School of Mathematics, Georgia Institute of Technology, Atlanta, 30332, Georgia, USA.
[2]Department of Mathematics and Statistics, Georgetown University, Washington, 20057, DC, USA.

*Corresponding author(s). E-mail(s): f.shirani@gatech.edu;
Contributing authors: Judith.Miller@georgetown.edu;

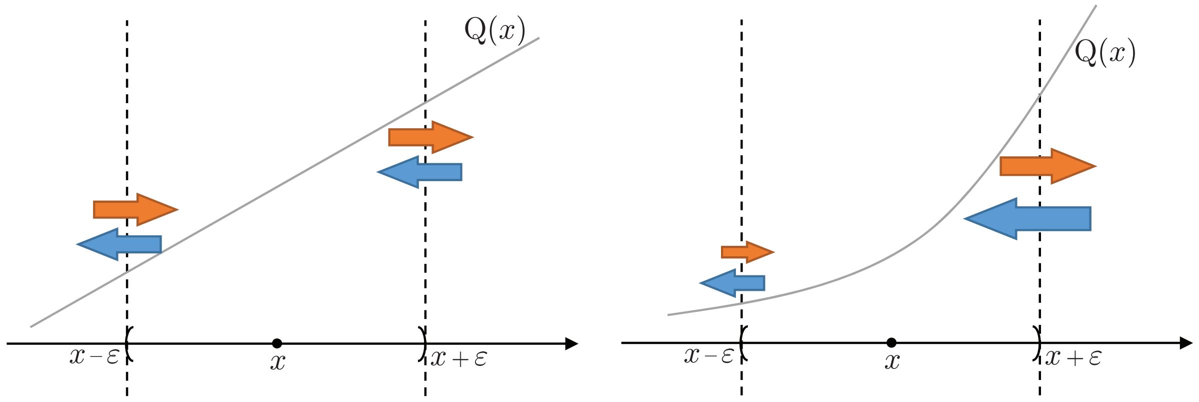

(a) **(b)**

Fig. S1: Effects of divergence in (perceived) environmental gradient on the mean trait value of a local population. We consider a local population located at a small neighborhood $(x - \varepsilon, x + \varepsilon)$ of a habitat point x. To illustrate changes in the trait mean that are purely caused by divergence in the perceived environmental gradient, that is the effects of the term (14b) in Equation (14) of the main text, we assume that the population density n, the trait variance v, and the optimal dispersal propensity A take constant values over a sufficiently large neighborhood of x, including $(x - \varepsilon, x + \varepsilon)$. Moreover, we assume that the dispersal rate of the individuals is independent of the magnitude of their phenotype-environment mismatch. Only the sign of the mismatch will determine whether they move in the direction or in the opposite direction of the environmental gradient. For simplicity, we further assume that the perceived gradient $\widetilde{\nabla_x \mathrm{Q}}$ is equal to the actual gradient $\nabla_x \mathrm{Q}$. As a result, the directed (optimal) dispersal rate will only be controlled by the magnitude of the environmental gradient. In each graph, the flow of individuals with higher phenotype value into and out of the ε-neighborhood is shown by red arrows. The blue arrows show the flow of lower phenotype values. Larger arrows indicate higher dispersal (flow) rates. The trait optimum $\mathrm{Q}(x)$ is increasing in x. As a result, the internal individuals that choose to move out of the neighborhood through the right boundary have larger phenotype values than the external individuals that choose to move into the neighborhood through this boundary. The opposite holds at the left boundary. In (a), the environmental gradient is constant, and we have $\mathrm{div}\left(\widetilde{\nabla_x \mathrm{Q}(x)}\right) = 0$. As a result, the inward and outward dispersal rates are equal at both boundaries, and their net effect does not change the mean trait value of the local population. In (b), the environmental gradient is increasing, and we have $\mathrm{div}\left(\widetilde{\nabla_x \mathrm{Q}(x)}\right) > 0$. As a result, the internal individuals that move out through the right boundary perceive a lower environmental gradient than the gradient perceived by external individuals that move in. That means, the outward dispersal rate at the right boundary is lower than the inward rate. The opposite holds at the left boundary. Moreover, since the environmental gradient at the right boundary is higher than the gradient at the lower boundary, the overall dispersal rate at the right boundary is higher than the rate at the left boundary. Consequently, as the arrows indicate, the local population loses more of its larger-valued phenotypes than it gains, and receives more of smaller-valued phenotypes than it loses. This leads to a reduction in the mean trait value of the local population due to the positive divergence in the environmental gradient.

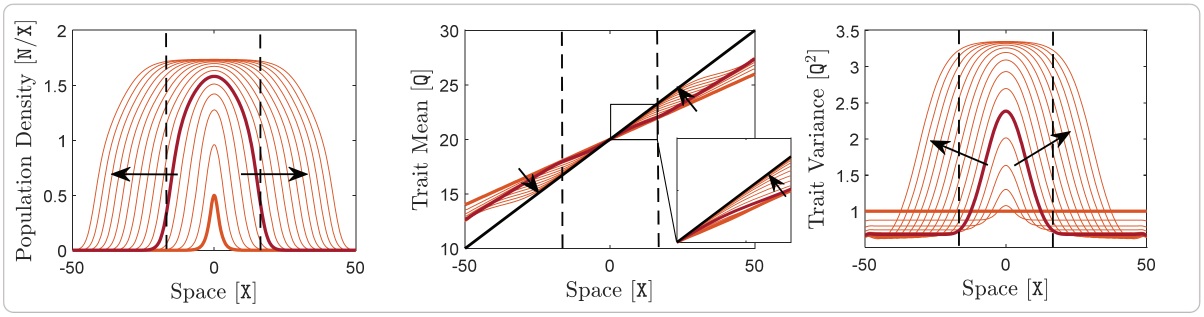

(a)

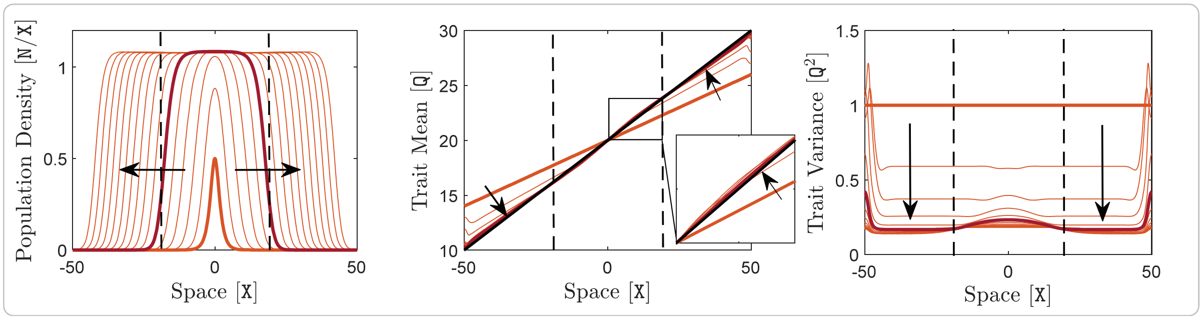

(b)

Fig. S2: Adaptive range dynamics of a species with specialist individuals in a one-dimensional habitat. Here, m $=1$ and A(x) is constant, taking different values in panels (a) and (b). The phenotype utilization variance takes the small value of V $=1$ $\mathtt{Q}^2$. The rest of the model parameters, including the environmental gradient, take their typical values given in Table 1 of of the main text. Panel (a) shows the range expansion dynamics of a species without optimal dispersal, A $=0$ $\mathtt{X}^2/\mathtt{T}$, whereas panel (b) shows the range expansion dynamics of a species with strong optimal dispersal, A $=10$ $\mathtt{X}^2/\mathtt{T}$. In all graphs, including the insets of trait mean graphs, curves are shown at every 1 T and a sample curve at $t=5$ T is highlighted in red. The same description as given in Figure 1 of the main text holds here for the quantities shown in each panel, the thick curves, arrows, and dashed lines.

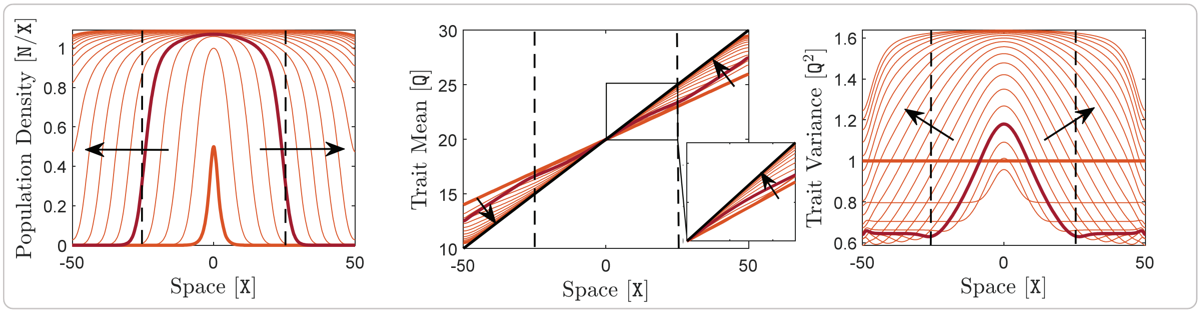

(a)

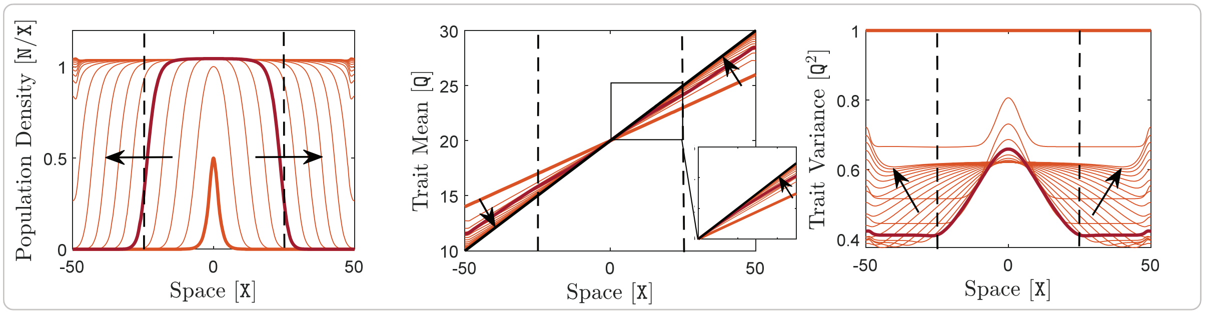

(b)

Fig. S3: Adaptive range dynamics of a species in a one-dimensional habitat with typical (shallow) environmental gradient. Here, m = 1, Q(x) is linear with the typical gradient of $\nabla_x \mathrm{Q} = 0.2\ \mathtt{Q}/\mathtt{X}$, and A($x$) is constant, taking different values in panels (a) and (b). The rest of the model parameters take their typical values given in Table 1 of the main text. Panel (a) shows the range expansion dynamics of a species without optimal dispersal, $\mathrm{A} = 0\ \mathtt{X}^2/\mathtt{T}$, whereas panel (b) shows the range expansion dynamics of a species with strong optimal dispersal, $\mathrm{A} = 10\ \mathtt{X}^2/\mathtt{T}$. In all graphs, including the insets of trait mean graphs, curves are shown at every 2 $\mathtt{T}$. The same description as given in Figure 1 of the main text holds here for the curves, arrows, and dashed lines.

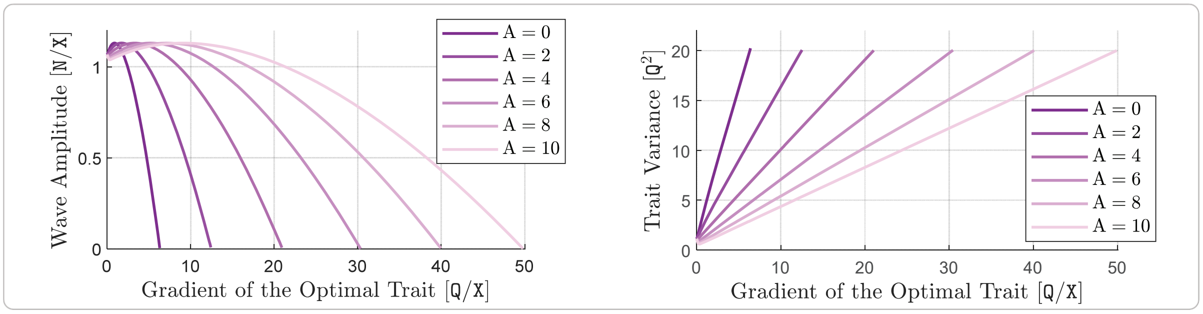

(a)

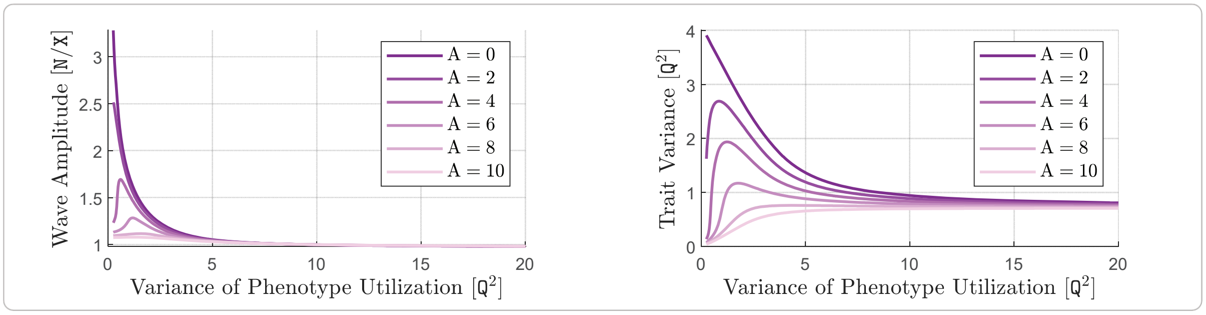

(b)

Fig. S4: Effects of optimal dispersal on range expansion waves and maximum intraspecific trait variance of a species. The graphs shown in this figure directly correspond to those shown in Figure 2 of the manuscript, with the only difference being that here the curves are computed more accurately based on the spatially homogeneous equilibrium of the model; see our previous work for details of such analysis (Shirani and Miller, 2022, Sect. 4.2). Specifically, $\mathrm{m} = 1$, and $\mathrm{A}(x)$ takes six different constant values in the range from $0\ \mathtt{X}^2/\mathtt{T}$ to $10\ \mathtt{X}^2/\mathtt{T}$. The trait optimum $\mathrm{Q}(x)$ is linear in x, with variable gradient in panel (a) and the constant gradient of $\nabla_x \mathrm{Q} = 0.2\ \mathtt{Q}/\mathtt{X}$ in panel (b). The phenotype utilization variance takes the constant value $\mathrm{V} = 4\ \mathtt{Q}^2$ in panel (a), and is variable in panel (b). The rest of the model parameters take their typical values given in Table 1 of the main text. In each panel, variations in the amplitude of the traveling waves are shown on the left and variations in the maximum intraspecific trait variance is shown on the right. Panel (a) shows the effects of different levels of optimal dispersal at different values of the environmental gradient $\nabla_x \mathrm{Q}$. Panel (b) shows the effects of different levels of optimal dispersal at different values of the individuals' phenotype utilization variance V.

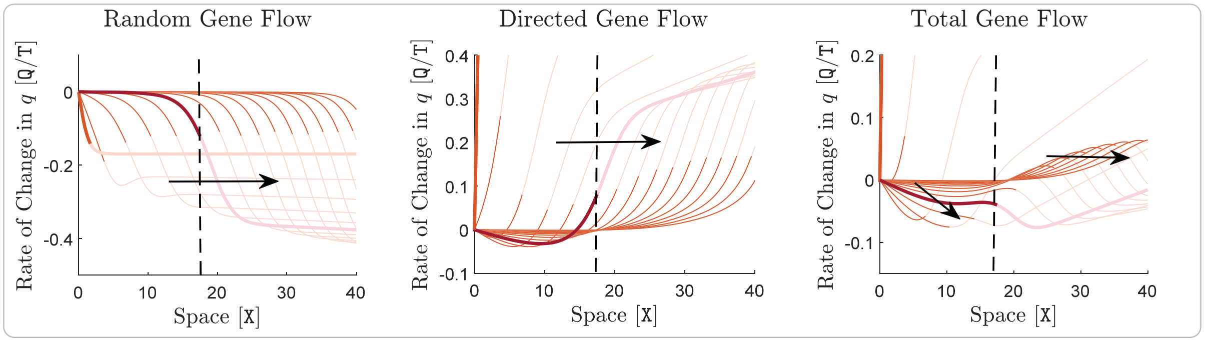

Fig. S5: Effects of gene flow on local adaption of a species. Contribution of random gene flow to the rate of change of the trait mean, $\partial_t q$, is shown on the left, contribution of directed gene flow generated by optimal dispersal is shown in the middle, and contribution of the total gene flow is shown on the right. The curves in each graph are calculated using the components of the trait mean equation as described in Figure 3 of the main text. The graphs correspond to the same simulation of species' range dynamics given in Figure S2b, that is, when $\mathrm{V} = 1\,\mathtt{Q}^2$ and $\mathrm{A} = 10\,\mathtt{X}^2/\mathtt{T}$. In all graphs, the evolution of the computed curves are shown only on the right half of the habitat. The curves extend symmetrically about the origin to the left half of the habitat. Moreover, the portion of each curve that lies outside the effective range of the species, that means over the regions where the population density rapidly vanishes to zero, has been made transparent. The descriptions of the time increments, highlighted curves, arrows, and dashed lines are the same as those provided in Figure S2b.

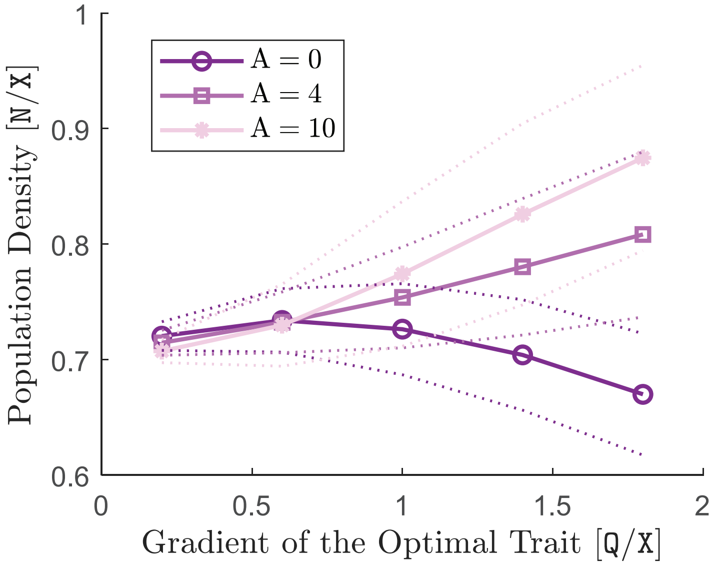

Fig. S6: Steady-state mean population density of a species under periodic abrupt fluctuations in the environmental trait optimum. The simulation setup is the same as that of Figure 5a in the main text, with the only difference being that the gradient of the optimal trait and A are made variable here. At each value of the gradient and each value of A, the simulation is run for a sufficiently long period of time so that the amplitude of fluctuations in the population density reaches a steady-state. The minimum and maximum values of such periodic fluctuations (peaks of the blue and red curves as shown in Figures 5a and 5b in the main text) are calculated near the end of the simulation, and are plotted by (interpolated) dotted lines. The average value of the fluctuations is shown by different markers for each value of A, along with an interpolated solid line.

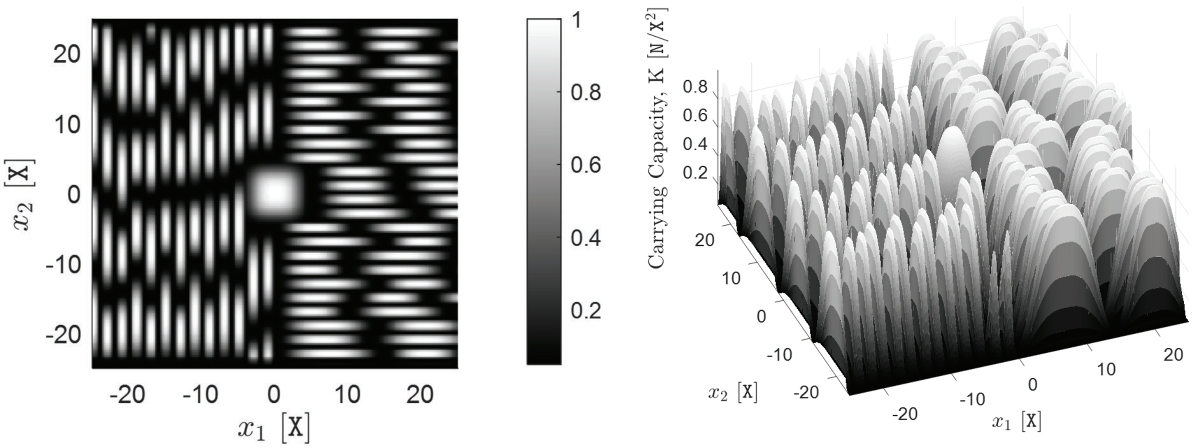

Fig. S7: Carrying capacity of the two-dimensional fragmented habitat. The spatial pattern of the carrying capacity $\mathrm{K}(x)$ used in the simulation of the two-dimensional fragmented habitat of Figure 7 in the main text. The parameter K takes its maximum value of $1\,\mathtt{N}/\mathtt{X}^2$ at the center of each patch, and its minimum value of $0.05\,\mathtt{N}/\mathtt{X}^2$ at the borderlines between the patches.

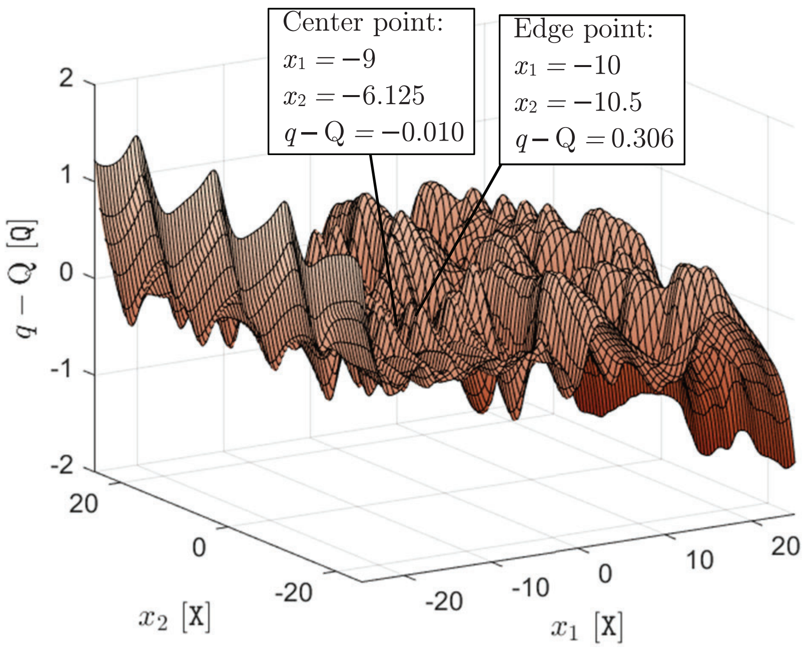

(a)

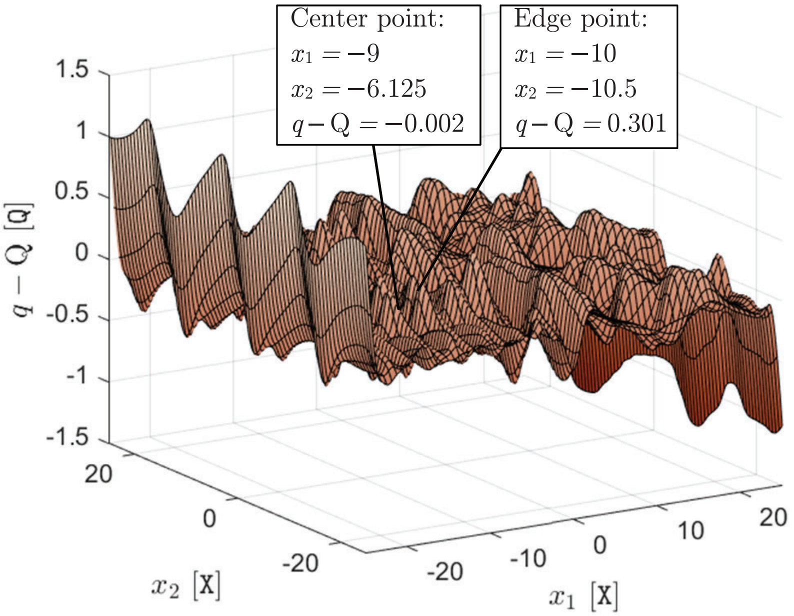

(b)

Fig. S8: Phenotype-environment mismatch of a species in a fragmented habitat. The results shown here correspond to the same simulation results shown in Figure 7 of the main text, for a species that spreads over a fragmented two-dimensional habitat. In each graph, the approximate steady-state profile of the phenotype-environment mismatch $q - \mathrm{Q}$ obtained at $t = 40\,\mathtt{T}$ is shown. Graph (a) shows the mismatch for the species with $\mathrm{A} = 0\,\mathtt{X}^2/\mathtt{T}$ (upper panel of Figure 7), and graph (b) shows the mismatch for the species with $\mathrm{A} = 10\,\mathtt{X}^2/\mathtt{T}$ (lower panel of Figure 7). Two samples of the exact values of the mismatch, one at the center of a patch and the other at its edge, are also explicitly specified in the graphs.

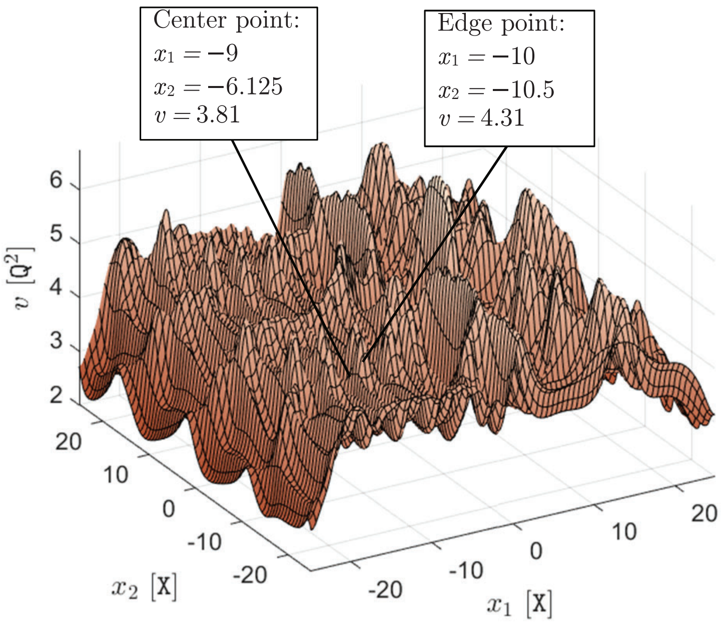

(a)

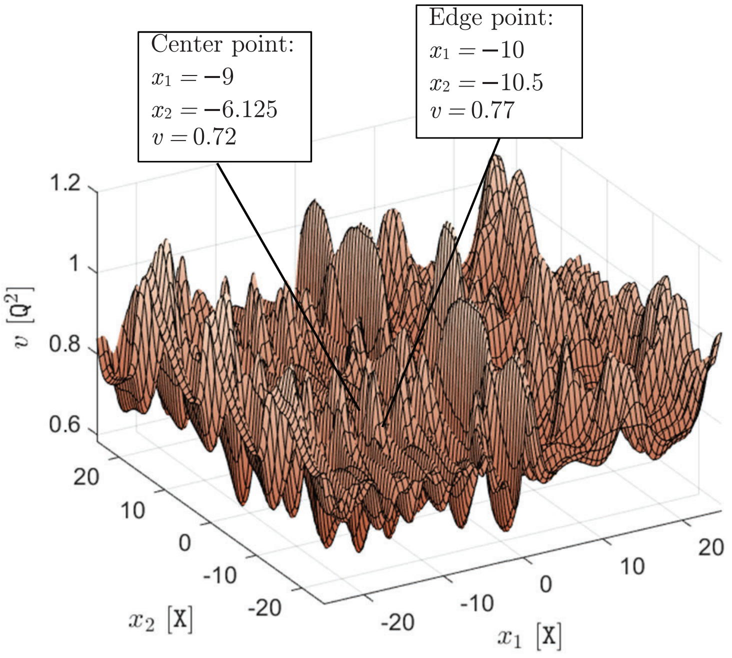

(b)

Fig. S9: Intraspecific trait variance of a species in a fragmented habitat. The results shown here correspond to the same simulation results shown in Figure 7 of the main text, for a species that spreads over a fragmented two-dimensional habitat. In each graph, the approximate steady-state profile of the intraspecific trait variance v obtained at $t = 40\,\mathtt{T}$ is shown. Graph (a) shows the trait variance for the species with $\mathrm{A} = 0\,\mathtt{X}^2/\mathtt{T}$ (upper panel of Figure 7), and graph (b) shows the trait variance for the species with $\mathrm{A} = 10\,\mathtt{X}^2/\mathtt{T}$ (lower panel of Figure 7). Two samples of the exact values of the trait variance, one at the center of a patch and the other at its edge, are also explicitly specified in the graphs.

References

Shirani, F., Miller, J.R.: Competition, trait variance dynamics, and the evolution of a species' range. Bulletin of Mathematical Biology **84**(3), 37 (2022) https://doi.org/10.1007/s11538-022-00990-z
